# Should schizoaffective disorder be diagnosed cross-sectionally (ICD-11) instead of longitudinally (DSM-5)? CON

**DOI:** 10.1192/j.eurpsy.2021.38

**Published:** 2021-08-13

**Authors:** I. Melle

**Affiliations:** Mental Health and Addiction, Institute of clinical medicine, University of Oslo, Oslo, Norway

## Abstract

There is a considerable overlap of psychotic and mood symptoms in patients with severe mental disorders, and a large proportion of patients meeting diagnostic criteria experience significant mood symptoms. The diagnosis of schizophrenia – schizoaffective type /schizoaffective disorder has been a part of the Diagnostic and statistical manual since the DSM-I. Even if the need for a diagnosis capturing this particular clinical picture obviously is needed, the agreement about when to use the diagnosis of schizoaffective disorder as well as the diagnostic reliability is low. There is an agreement that the diagnosis covers episodes with an overlap between a psychotic episode meeting the A criteria for schizophrenia and a major mood episode where the psychotic symptoms continue after the end of the mood symptoms. Given the prevalence of depression in schizophrenia, some episodes, however, meet the criteria for schizoaffective disorders while others do not. The DSM-5 thus argues that the pattern of overlapping symptoms should be present in the majority of episodes. Keeping the cross-sectional approach favoured by previous versions of the DSM and the ICD will here continue to spread diagnostic confusion.

**Disclosure:**

No significant relationships.

